# Assessment of Dairy Cattle Management System and Uses of Antimicrobials Among Smallholder Farmers in Korogwe District, Tanzania

**DOI:** 10.1155/vmi/8596479

**Published:** 2026-06-26

**Authors:** Goodluck Cleophas Mushi, Amina Ramadhani Issae, Hezron Emmanuel Nonga

**Affiliations:** ^1^ Department of Veterinary Medicine and Public Health, College of Veterinary Medicine and Biomedical Sciences, Sokoine University of Agriculture, P.O. Box 3021 Chuo Kikuu, Morogoro, Tanzania, suanet.ac.tz; ^2^ Korogwe District Council, P.O. Box 584, Korogwe, Tanzania; ^3^ Institute of Pest Management, Sokoine University of Agriculture, P.O. Box 3110, Morogoro, Tanzania, suanet.ac.tz

**Keywords:** antimicrobial use, cattle diseases, drug withdrawal period, livestock production, milk

## Abstract

Good livestock husbandry practices associated with disease preventive measures contribute to improved production of quality and safe livestock products. Poor livestock management, climatic conditions, and diseases are among the catalysts to inappropriate use of veterinary drugs, particularly antimicrobials, which have significant public health risk. The purpose of this cross‐sectional study was to evaluate cattle management systems, livestock diseases and preventive measures, and the use of antimicrobials among smallholder dairy cattle farmers (*n* = 82) and other stakeholders (*n* = 9). Data were collected using structured questionnaires. Descriptive statistics summarized the data, and analysis was done using Statistical Package for the Social Sciences Version 25.0. Almost 55% of farmers managed their cattle under an intensive/semi‐intensive production system and practiced disease control measures by good husbandry practices (63%), vaccination (62%), and applied acaricides (49%). Nine cattle diseases were reported, dominated by vector‐borne diseases, particularly trypanosomiasis (59%) and tick‐borne diseases (49%). All farmers admitted using antimicrobials that were self‐administered (73.2%), and awareness of withdrawal periods was low (45.1%). Significant predictors of antimicrobial use (AMU) included sex of the farmer, production system, and access to training (*p* < 0.05). AMU was significantly higher among female farmers (odds ratio [OR] = 9.71) and those with access to training (OR = 8.66). Conversely, farmers utilizing extensive grazing systems had 92.1% lower odds of AMU compared to those under intensive/semi‐intensive systems (OR = 0.08). Antimicrobial misuse in Korogwe District is driven by socio‐demographic factors, production systems, and limited access to training, with low awareness of withdrawal periods increasing the risk of drug residues in milk. Strengthened extension services, improved disease prevention practices, and stricter compliance with withdrawal periods are essential to reduce these risks.

## 1. Introduction

Livestock production remains a cornerstone of rural livelihoods, food security, and the national economy across many low‐ and middle‐income countries. In Tanzania, smallholder and pastoral systems dominate, ranging from extensive grazing to semi‐intensive and intensive dairy production [1–3]. Smallholder farming contributes significantly to household income and nutrition and the dairy industry [2, 4]. However, cattle farming in Tanzania faces persistent challenges such as feed scarcity, climate variability, and livestock diseases [1, 2]. Vector‐borne diseases, including East Coast fever (ECF), anaplasmosis, babesiosis, and trypanosomiasis, are endemic. Other major livestock diseases in Tanzania include contagious bovine pleuropneumonia (CBPP), foot and mouth disease (FMD), lumpy skin disease (LSD), mastitis, and infertility [5, 6]. The livestock diseases are often exacerbated by poor husbandry practices, climatic pressures, and limited access to veterinary extension services.

Nonetheless, the government, farmers, and other stakeholders implement disease control measures, including the uses of acaricides and vaccination programs [7]. Between 2003 and 2014, the government built and renovated 1274 dip tanks and supplied around 1.2 million liters of subsidized acaricides to farmers. Similarly, between 2018 and 2024, the government implemented another dipping campaign, which led to 3100 functional dip tanks and a supply of 184,859.79 L of subsidized acaricides, consequently resulting in a significant decrease of vectors and vector‐borne diseases [8, 9]. Nevertheless, between 2025 and 2029, the government is implementing a vaccination program against priority livestock diseases worth 217 billion shillings (91 million USD), which is expected to eradicate some of the diseases like CBPP, Peste des Petits Ruminants (PPR), and CCPP and create FMD‐free compartments [10].

Routine use of veterinary drugs for treatment and prophylaxis in livestock is common. Farmers are advised to use prophylactic drugs like anthelmintics and trypanocides in ruminants at 3‐month intervals [11]. Generally, animal health experts and some farmers use antimicrobials for prophylaxis and growth promoters [12, 13]. Sometimes, antimicrobials are used for the treatment of ill health regardless of diagnosis, which always leads to misuse or indiscriminate uses [12, 14, 15]. However, misuse of antimicrobials is driven by high disease burdens, limited availability of diagnostics, economic pressures to maintain herd productivity, and easy access to over‐the‐counter antimicrobials [12, 14, 15]. Inadequate knowledge on antimicrobial dosages, routes of administration, and withdrawal periods further increases the risk of inappropriate use that always threatens food safety and public health [12, 16]. Studies show that farmers’ decisions around antimicrobial use (AMU) are not determined solely by disease occurrence; rather, they are influenced by financial limitations, unequal access to trustworthy animal health information, cultural beliefs, and reliance on informal community advice networks [16, 17].

Korogwe is among the districts with farmers that keep indigenous and dairy cattle under extensive and semi‐intensive grazing systems with similar disease challenges. Apart from applying some disease preventive measures in livestock, uses of antimicrobials are practiced since there are 24 veterinary shops in the district. Farmers and animal health experts access the antimicrobials and other veterinary drugs and use them for different purposes [17, 18]. Meanwhile, there has been no study on dairy cattle management systems, livestock disease, livestock disease preventive measures, AMUs, knowledge on drug residues, and withdrawal periods among the smallholder cattle farmers in Korogwe, Tanzania. The aim of this study was to assess the cattle management systems, livestock diseases and preventive measures, and the uses of antimicrobials among smallholder dairy cattle farmers, veterinary shop sellers, livestock field officers (LFOs), and operators of milk collection centers.

## 2. Materials and Methods

### 2.1. Study Area and Livestock Production

**FIGURE 1:**
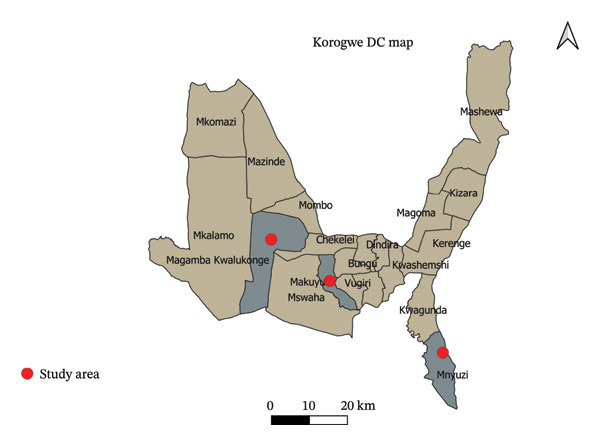
The map showing the areas where the study was conducted in Korogwe District, involving three study wards shown by red dots.

The study was conducted in Korogwe District Council (latitude ∼ 5°00′ S to 5°15′ S; longitude ∼ 38°00′ *E* to 38°45′ E) in Tanga Region, Tanzania (Figure 1). Administratively, Korogwe District Council has 29 wards with an estimated human population of 272,870 [19]. Climatically, the district experiences a bimodal rainfall pattern, with a long rainy season occurring from March to May and the short rainy season from October to December, while dry seasons extend from June to September and January to February. Annual rainfall ranges between 800 mm and 1400 mm, with temperatures varying from 18 °C to 32 °C.

Korogwe District Council has a population of cattle estimated at 154,332, among which approximately 6050 are exotic dairy breeds (mainly Friesian and Ayrshire crosses) raised under intensive and semi‐intensive production. The indigenous breed, dominated by the Tanzania Short Horn Zebu, is grazed under extensive systems and is owned by pastoral and agro‐pastoral communities [3]. Most of the milk produced is distributed through informal channels involving direct household consumption, local vendors, smallholder kiosks, restaurants, and hotels. Despite this, a small portion of the milk enters the formal marketing system via three milk‐collection and cooling centers located in Hale Ward, Makuyuni Ward, and Magamba Kwalukonge Ward. Korogwe District was selected for study due to its diverse dairy cattle population and production systems, providing a representative setting for assessing livestock management practices, milk productivity, and marketing dynamics in Tanzania.

### 2.2. Study Design and Participants

This cross‐sectional study was conducted between September and November 2025 using a multistage sampling approach to select respondents along the dairy value chain. Three wards, namely Hale, Magamba Kwalukonge, and Makuyuni, were purposively selected based on dairy farming intensity and the presence of active milk collection centers. A total of 14 villages (seven in Hale, three in Magamba Kwalukonge, and four in Makuyuni) were randomly selected via a lottery method. A verified sampling frame was developed in collaboration with the district livestock and fisheries officer (DLFO) and ward LFOs to identify potential participants. The inclusion criteria for smallholder cattle farmers were owning at least one lactating cow at the time of sampling, residing within the study area, being available during the data collection period, and voluntarily providing informed consent. For the key informants, comprising three LFOs, three milk collection center managers, and three agrovet sellers, inclusion was based on their active professional role in the dairy value chain and a minimum of 1 year of experience in the study area.

### 2.3. Data Collection

Data were collected using group‐specific semistructured questionnaires from 82 smallholder cattle farmers, three LFOs, three veterinary shop sellers, and three milk collection center managers (Supporting Information). Each questionnaire featured domain‐specific modules consisting of closed and open‐ended questions designed to capture socio‐demographic information (six questions), farming systems, livestock diseases and their control measures (nine questions), and other nine questions on uses of antimicrobial in cattle, awareness of potential health risks associated with antimicrobials residues, and compliance with withdrawal periods (Supporting Information).

For analytical purposes, respondents’ ages were grouped as youth (< 45 years) and elders (≥ 45 years). Cattle ownership was assessed based on the number of cattle kept per household. Herd size was categorized as < 20 cattle (small herds) and ≥ 20 cattle (large herds). This approach follows livestock and smallholder dairy research from Tanzania and East Africa, which frequently uses context‐specific herd‐size or production‐system classifications rather than a single universal numeric cutoff [3, 20, 21]. Prior to the survey, the questionnaire was pretested among a small group of smallholder cattle farmers outside the study area in Korogwe District to assess clarity, relevance, and logical flow.

### 2.4. Data Management and Analysis

The data obtained from the completed questionnaires were first coded and entered in Microsoft Excel spreadsheets for cleaning and validation to ensure completeness and consistency. The cleaned dataset was then exported to the Statistical Package for the Social Sciences (SPSS) Version 25.0 for statistical analysis. Descriptive statistics, including frequencies, and percentages were computed to summarize the socio‐demographic characteristics of respondents and to describe patterns of AMU and livestock management practices among different stakeholder groups. The dependent variable for this study, AMU, was obtained through self‐reported survey data and coded as a binary outcome (1 = use; 0 = non‐use). Statistically, this variable follows a Bernoulli distribution. Due to its dichotomous nature and non‐normal distribution, a logit link function was applied within the regression framework.

Inferential statistical analysis was subsequently performed to explore associations between selected independent variables and AMU. Specifically, binary logistic regression analysis was employed to identify significant predictors at a 95% confidence interval (CI). Variables with *p* < 0.05 were considered statistically significant. The results were presented in tabular form and interpreted with corresponding odds ratios (ORs) to quantify the strength and direction of associations. The qualitative data from key informant interviews were analyzed using thematic and content analysis following the systematic framework described by Braun and Clarke [22]. This involved a recursive process of data familiarization, initial code generation, and the aggregation of codes into meaningful themes related to AMU practices.

## 3. Results

### 3.1. Socio‐Demographic Characteristics of Respondents

The socio‐demographic characteristics of the respondents are summarized in Table 1. A total of 82 smallholder dairy farmers participated in the study; the majority were males (*n* = 66; 80.5%). The mean age of respondents was 52.12 ± 10.35 years. The majority of farmers (*n* = 61; 75.3%) had primary education, and most farmers were from Hale Ward (*n* = 39; 47.6%), reflecting the concentration of cattle farming activities in this area.

**TABLE 1:** Socio‐demographic information of smallholder dairy cattle farmers in Korogwe District (*n* = 82).

Variables	Category	Frequency	Percentage (%)
Sex	Male	66	80.5
	Female	16	19.5

Age	Youth (< 45 years)	33	40.24
	Elder (≥ 45 years)	49	59.76

Education	Nonformal	1	1.2
	Primary	61	75.3
	Secondary	15	18.3
	College	5	6.1

Ward	Hale	39	47.6
	Magamba	25	30.5
	Makuyuni	18	22.0

### 3.2. Herd Structure of Smallholder Dairy Cattle Farms

The herd composition and management practices of the surveyed farms are summarized in Table 2. The dominant production system was intensive/semi‐intensive grazing (*n* = 45; 54.9%), and most farmers (*n* = 66; 80.5%) kept Friesian crosses. Across 82 households, a total of 207 were lactating cows, and the average milk production was 5.95 ± 4.98 L per cow per day. Overall herd sizes ranged from 1 to 25 cattle per household.

**TABLE 2:** Herd structure of smallholder dairy cattle farms in Korogwe District.

Variables	Category	Frequency	Percentage (%)
Number of cattle kept	Less than 20	78	95.1
	More than 20	4	4.9

Breed of cattle	Friesian cross	66	80.5
	Jersey cross	1	1.2
	Ayrshire cross	15	18.3

Farming system	Intensive/semi‐ intensive	45	54.9
	Extensive	37	45.1

Cow status	Lactating	207	38.6
	Non‐lactating	330	61.5

*Note:* Percentages for herd composition variables (breed types and cow status) are calculated using the total number of animals within each category, not the number of households (*N* = 82).

### 3.3. Milk Marketing Practices Among Smallholder Dairy Cattle Farmers

The milk marketing practices among smallholder dairy cattle farmers are summarized in Table 3. Most farmers (*n* = 79; 96.3%) reported selling milk mostly to neighbors (*n* = 53; 64.6%). The remainder sold milk through informal channels such as household buyers, vendors, neighbors, and local kiosks.

**TABLE 3:** Milk marketing practices among smallholder dairy cattle farmers.

Variables	Category	Frequency	Percentage (%)
Do you sell milk?	Yes	79	96.3
	No	3	3.7

Milk selling points	Households (neighbors)	53	64.6
	Milk collection center	33	27.3
	Vendors	20	16.5
	Restaurants/kiosks	15	12.4

### 3.4. Livestock Diseases and Their Control

**FIGURE 2:**
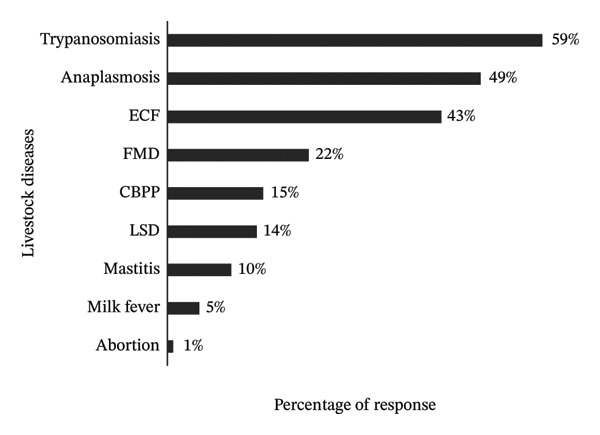
Diseases of cattle reported by the smallholder dairy cattle farmers in the study area. Note that ECF is East Coast fever, contagious bovine pleuropneumonia (CBPP), foot and mouth disease (FMD), and lumpy skin disease (LSD).

The reported livestock diseases that affect dairy cattle in the study area are presented in Figure 2. Nine cattle diseases were reported to be dominated by vector‐borne diseases, particularly trypanosomiasis (59%) and tick‐borne diseases (49%). The disease control measures reported by smallholder dairy farmers included good husbandry practices (63%), vaccination (62%), and use of acaricides (49%).

### 3.5. Uses of Antimicrobials in Dairy Cattle in Korogwe District

**FIGURE 3:**
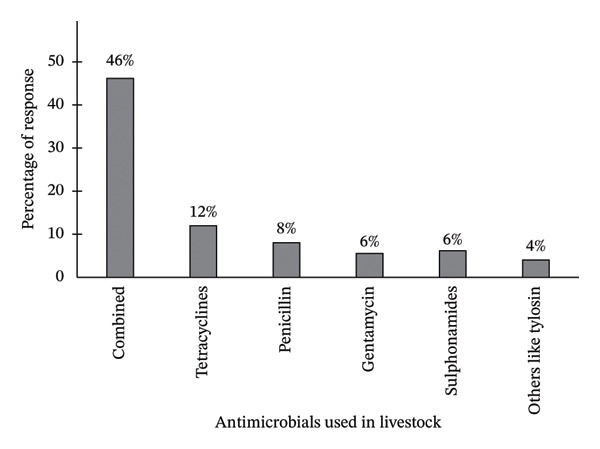
Types of antimicrobials reported to be used in cattle.

The distribution of AMU among smallholder dairy farmers in the study area is presented in Figure 3. All smallholder dairy farmers in the study area reported using antimicrobials for disease prevention and treatment. Over half (56.1%) used combinations of antimicrobials, with tetracyclines being the most preferred. Table 4 contains information on milk from cows under antimicrobial treatment. Almost half (50.3%) of the smallholder dairy cattle farmers reported using antimicrobials for therapeutic purposes, which were self‐administered to cattle. Up to 51.2% of the respondents reported consuming or selling milk from cows under treatment since only 45.1% were aware of drug withdrawal periods. Furthermore, most farmers (91.5%) had not received any formal training on the proper use of veterinary drugs.

**Table 4:** Reponses on milk from cows under antimicrobial treatment.

Factors	Category	Frequency	Percentage (%)
Do you use antimicrobials?	Yes	62	75.6
	No	20	24.4

What are the reasons for use (*n* = 163)	Treatment	82	50.3
	Prevention	81	49.7

Who administers the antimicrobials?	Self	60	73.2
	Agrovet dealer	2	2.4
	Livestock expert	18	22.0
	Others (experienced neighbor farmers)	2	2.4

Do you consume or sell milk from a cow under antimicrobial treatment?	Yes	42	51.2
	No	40	48.8

Are you aware of the antimicrobial withdrawal period?	Yes	37	45.1
	No	45	54.9

Do you observe the withdrawal period before milking? (*n* = 37)	Sometimes	19	51.4
	Never	12	32.4
	Always	6	16.2

Have you ever been trained on drug use in animals	Yes	7	8.5
	No	75	91.5

What do you do with milk during withdrawal period (*n* = 37)	Discard	6	16.2
	Use for household	9	24.3
	Feed to calves	12	32.4
	Sell	10	27.0

*Note:* Percentages for antimicrobial usage reasons are calculated based on total use events (*n* = 163); percentages for milk use during treatment are based on total use events (*n* = 37); all other percentages are calculated based on the total number of respondents (*n* = 82).

### 3.6. Possible Factors for AMU in Dairy Cattle

Binary logistic regression results on the factors for AMU are presented in Table 5. The model’s goodness of fit was evaluated using the Hosmer and Lemeshow test, which yielded a statistically insignificant result (*X* ^2^ = 22.879; *p* = 0.243). The results showed that sex, type of management, and access to training were significant factors for AMU in the study area (*p* < 0.05). Gender was significantly associated with AMU; female farmers were 9.71 times more likely to use antimicrobials compared to their male counterparts (OR = 9.71). In addition, the management system significantly influenced use, with farmers practicing extensive grazing having 92.1% lower odds of using antimicrobials than those under intensive/semi‐intensive systems (OR = 0.08). Moreover, access to training had a positive influence, with trained farmers being 8.7 times more likely to use antimicrobials than those without (OR = 8.66).

**TABLE 5:** Possible factors for antimicrobial use in dairy cattle.

Factor	Category	Coefficient (B)	SE	Wald	*p*‐value	Odd ratio/Exp (B)	95% CI for OR
Sex	Male (Ref.)	0				1	
	Female	2.278	0.962	5.608	0.018^∗^	9.71	(1.47–64.1)

Age group	Other age groups (Ref.)	0				1	
	30–40	0.749	0.395	3.587	0.058	2.11	(0.975–4.583)

Education level	No formal education (Ref.)	0				1	
	College	15.739	0.401	0.000	1.000	0.68	(0.310–1.491)
	Secondary	−0.294	1.674	0.031	0.861	0.75	(0.028–19.852)
	Primary	0.586	1.927	0.093	0.761	1.80	(0.041–78.361)

Management system	Extensive (Ref.)	0				1	
	Intensive/semi‐intensive	−1.464	1.261	1.347	0.246	0.23	(0.019–2.738)
	Extensive	−2.544	1.285	3.918	0.048^∗^	0.08	(0.006–0.985)

Milk market	No (Ref.)	0				1	
	Yes	0.090	1.813	0.002	0.960	1.10	(0.031–38.257)

Ward	Other words (Ref.)	0				1	
	Hale	0.821	0.983	0.697	0.404	2.27	(0.331–15.584)
	Makuyuni/M/Kwalukonge	−0.591	0.885	0.446	0.504	0.55	(0.098–3.132)

No. of cattle	Continuous	−0.001	0.048	0.001	0.980	0.10	(0.910–1.097)

Administer	None/Other (Ref.)	0				1	
	Livestock expert	−1.137	1.739	0.428	0.513	0.32	(0.011–9.658)
	Agrovet dealer	2.062	0.254	0.000	0.999	7.86	(4.777–12.939)
	Self	0.500	0.834	0.360	0.549	1.65	(0.321–8.465)

Access to training	No (Ref.)	0				1	
	Yes	2.159	0.951	5.151	0.023^∗^	8.66	(1.341–55.975)

Disease control	None (Ref.)	0				1	
	Dipping	−1.683	0.962	3.058	0.080	0.19	(0.028–1.225)
	Vaccination	0.000	0.822	0.000	1.000	1.00	(0.200–4.998)
	Do nothing	1.405	1.005	1.956	0.162	4.08	(0.567–29.288)

Withdrawal period	No (Ref.)	0				1	
	Yes	−1.331	0.845	2.479	0.115	0.26	(0.050–1.385)

Constant	Intercept	1.285	2.343	0.301	0.583	3.62	
	n	82					

^∗^Statistically significant at *P* < 0.05, SE—standard error, CI—confidence interval.

### 3.7. Results of Key Informant Interviewers

Table 6 shows the demographic information of the nine key informant respondents interviewed. The key informants included agrovet sellers (*n* = 3), LFOs (*n* = 3), and milk collection center managers (*n* = 3). Of the 9 key informants, seven were males and had either a certificate or diploma in Animal Health and Production.

**TABLE 6:** Demographic characteristics of key informants studied (*n* = 9).

Category of key informant	No.	Ward	Sex	Qualifications	Years of experience (years)
Agrovet shop sellers	1	Hale	Male	Secondary education	5
	2	Makuyuni	Male	Certificate in Animal Health and Production	3
	3	Magamba	Male	Certificate in Animal Health and Production	10

Livestock field officers	1	Hale	Female	Diploma in Animal Health and Production	3
	2	Makuyuni	Male	Diploma in Animal Health and Production	10
	3	Magamba	Male	Diploma in Animal Health and Production	6

Managers of milk collection centers	1	Hale	Male	Certificate in Animal Health and Production	1.5
	2	Makuyuni	Female	Secondary education	1
	3	Magamba	Male	Certificate in Animal Health and Production	2

The results from the Agrovet sellers show that antimicrobials, in particular tetracyclines, penicillin‐dihydrostreptomycin, sulfonamides, gentamicin, tylosin, and streptomycin, were the frequently sold veterinary drugs. All the veterinary drugs, including antimicrobials, were sold over the counter without requiring prescriptions. None of the Agrovet sellers reported instructing users of antimicrobials on the withdrawal period. The Agrovet sellers confessed to knowing the health risks of antibiotic residues in milk, and among others, they mentioned milk contamination, allergic reactions, bad smell in milk, interference with milk processing, and antimicrobial resistance. The Agrovet sellers reported getting training on prudent use of antimicrobials when they were at the colleges.

Information accrued from LFOs indicated that antimicrobials are frequently used in dairy cattle for treatment and prevention of diseases; they use them as first‐line drugs to take care of different health problems in animals. The LFOs further noted that oxytetracyclines, sulfonamides, penicillin‐dihydrostreptomycin, gentamicin, trypanocides, anthelmintics, mastitis tubes, OTC spray, wound powder, acaricides, and some vaccines were the veterinary drugs used in dairy cattle. All the LFOs confessed to giving instructions to farmers on the withdrawal period of milk from cows under antimicrobial treatments but were not sure how farmers comply, especially in the absence of structured monitoring for antimicrobial residues. They further reported not to conduct training/sensitization for farmers on drug use and withdrawal periods. There are no reported cases of drug residues in milk in Korogwe because there are no tests for residues in milk.

The managers of milk collection centers reported receiving milk from many regular suppliers and performed only basic quality tests like sensory tests like smell, color, and cleanliness of milk, lactometer tests, and alcohol tests. There is no routine test for antimicrobial residue apart from smell. The main suppliers of milk include the farmers and vendors. On average, each milk collection center collects 370 ± 546 L per day. The managers of milk collection centers were not sure as to whether the suppliers of milk had any kind of training on the risks of drug residues in milk. Occasionally, the milk collection centers get milk that fails the platform tests or milk reception tests, mostly abnormal smell, abnormal color, dirty milk, and lactometer tests. Upon rejection, the milk suppliers are always educated on how to supply good‐quality milk.

## 4. Discussion

This study assessed cattle management systems, livestock diseases, and the use of antimicrobials among smallholder dairy cattle farmers in Korogwe District. The findings indicate that socio‐demographic characteristics, farm management practices, and livestock diseases collectively influence AMUs. Understanding these factors is essential for developing effective interventions to promote prudent AMU and improve milk safety.

Dairy farming in Korogwe is predominantly male‐dominated (80.5%), reflecting cultural norms that position men as household heads and formal livestock owners. However, women undertake most routine husbandry tasks, including milking and daily animal care, which may foster more cautious antimicrobial handling. This pattern aligns with findings from Arusha, Kilimanjaro, Manyara, Tanga, and Morogoro, where women similarly manage daily herd activities despite men retaining formal ownership [16, 18]. Unlike earlier studies that highlight women’s limited authority over antimicrobial decisions, the Korogwe results indicate that their active, hands‐on involvement may enhance prudent AMU, suggesting a shared gendered labor structure across regions but a distinct local effect on antimicrobial practices. Most farmers in this study had only primary education (75.3%), likely due to limited access to secondary schooling, household economic constraints, and cultural norms in Tanzanian livestock‐keeping communities that prioritize early involvement in farming. This limited formal training may affect understanding of proper AMU. Similar patterns have been reported in Morogoro and Arusha**,** where farmers also rely heavily on experiential knowledge when making treatment decisions [23, 24]. Thus, the Korogwe findings are consistent with trends observed in Morogoro**,** although the extent to which experience compensates for limited education may vary across regions. Smallholder farms predominate in Korogwe due to limited land, capital, and access to veterinary services, reflecting the typical smallholder livestock systems in Tanzania, where most animals are maintained on small, family‐run holdings rather than commercial farms [4]. These findings align with other studies elsewhere that report many livestock keepers operate as smallholders, with production practices and herd sizes constrained by low capital, small landholdings, and restricted veterinary support [25, 26]. Collectively, these socio‐demographic patterns underscore how gender, age, education, and farm size interact to influence antimicrobial practices in smallholder dairy production.

Tetracyclines were the predominant antimicrobial used by farmers, likely due to their broad‐spectrum characteristics, affordability, and ready availability from local veterinary shops [14]. Key informant interviews confirmed that tetracyclines were the most frequently sold and routinely administered drugs. This finding mirrors other studies in Tanzania, which report that uses of tetracyclines in veterinary practices may be due to their easy accessibility, limited diagnostic capacity, and inadequate veterinary coverage [18, 27, 28]. There is an urgent need for stewardship programs, improved diagnostics, and training to support more responsible drug use.

Vector‐borne diseases, in particular trypanosomiasis, ECF, and anaplasmosis, were reported in Korogwe, which may be due to favorable conditions that support the survival and multiplication of tsetse flies and ticks [28, 29]. The endemicity of these diseases likely drives frequent AMU, as farmers attempt to manage recurrent infections [12, 14, 18, 20, 30]. Further, extensive grazing systems were associated with lower AMU among smallholder dairy farmers, likely because wider grazing areas and low animal densities reduce disease spread and sometimes farmers depend more on traditional treatment using medicinal plants and other remedies [20]. Confinement of cattle may create stress and increased chances for disease transmission that consequently lead to increased uses of antimicrobials [15, 16, 31, 32].

Further, the findings revealed that female farmers had significantly higher odds of using antimicrobials, which is likely attributable to the prevailing gender‐based roles within smallholder dairy management systems. In the Tanzanian context, women often assume primary responsibility for routine husbandry tasks, including feeding, cleaning, and health monitoring consequently uses of antimicrobials as previously reported [33]. Additionally, while training is intended to promote prudent use, it often increases a farmer’s ability to recognize diseases, which may lead to a higher frequency of AMUs as was observed in the current study [16]. Differently, a study by Higham et al. [34] in Kenya reported training reduces AMU in livestock. The difference may stem from the content of the training provided in the study area, which might focus more on disease recognition and treatment rather than prevention and antimicrobial stewardship.

Poor adherence to the antimicrobial withdrawal period was observed in Korogwe and was probably associated with low literacy levels, inadequate record‐keeping, limited access to veterinary guidance, and economic pressure to sell milk during treatment periods. Key informants further reported that although smallholder dairy farmers had received training on the withdrawal period, compliance remained low. These findings closely mirror patterns reported in other studies [16, 18].

Farmers primarily relied on hygiene‐based practices to prevent diseases, a pattern driven by the low cost of these measures, financial constraints, their relative ease of implementation, and their grounding in traditional knowledge already familiar to farmers. Limited access to veterinary extension services may further reinforce this reliance. Consequently, the adoption of vaccination, vector control, and other biosecurity measures remained low. While hygiene is essential, overreliance on this approach alone leaves herds vulnerable to infections, driving higher AMU. Similar patterns have been reported in Arusha and Manyara [17], but Korogwe shows an even greater dependence on hygiene, likely due to limited veterinary access and financial constraints.

## 5. Conclusion and Recommendations

This study shows that poor livestock management predisposes cattle to diseases that lead to the uses of antimicrobials, particularly tetracyclines. Farmer sex, production system, and access to training significantly predict AMUs in cattle. Low awareness and poor compliance with withdrawal periods increase the chance of supplying milk with antimicrobial residues. To improve milk safety and protect public health, it is essential to strengthen veterinary extension services, enforce withdrawal‐period regulations, promote responsible AMU, and implement regular residue testing programs. Building farmers’ capacity through training on safe antimicrobial administration, preventive herd health, and vector control, alongside enhancing dairy value chain practices such as “reject‐and‐educate” protocols at milk collection centers, will further reduce antimicrobial misuse and residue risks.

## 6. Study Limitations

This study was cross‐sectional, focused on three wards within Korogwe District, and involved a moderately small sample size; therefore, the findings may not be fully generalizable to all smallholder dairy systems in Tanzania. Data collection relied exclusively on self‐reported responses obtained through questionnaires and key informant interviews, which may be subject to recall bias, misreporting, social desirability bias, and self‐reporting errors, particularly regarding drug use behaviors and adherence to withdrawal periods. Additionally, the study’s limited geographic coverage may restrict the broader applicability of the results. Despite these limitations, the findings offer important insights into AMU drivers and practices within smallholder dairy farms.

## Author Contributions

Mushi, Goodluck Cleophas: conceptualizing of research ideas, writing of proposal, data collection, and drafting of manuscript; Issae, Amina Ramadhani: supervision of research, data analysis and interpretation, and drafting of manuscript; Nonga, Hezron Emmanuel: supervision of the whole research, data analysis and interpretation, review, and perfection of manuscript. All the authors have read and agreed to the published version of the manuscript.

## Funding

No funding was received for this research.

## Ethics Statement

Ethical approval and research authorization for this study were obtained from Sokoine University of Agriculture (SUA) under permit number REF NO. RSUA/ADM/R.1/8/1511, The Tanzania Livestock Research Institute (TALIRI) under permit number REF NO. TLRI/CC.21/075**.** In addition, permission to conduct the research within local government authorities was granted by the President’s Office—Regional Administration and Local Government (PO‐RALG) under permit number REF NO. AB.307/323/01″R”/493. Administrative clearance to carry out field activities in Korogwe District Council was provided by the Korogwe District Executive Director. Prior to data collection, all participants were informed about the objectives and significance of the study, and verbal consent was obtained to ensure voluntary participation. Confidentiality and anonymity of respondents were maintained throughout the study, and all data collected were used exclusively for research purposes in accordance with ethical research standards.

## Conflicts of Interest

The authors declare no conflicts of interest.

## Supporting Information

Additional supporting information can be found online in the Supporting Information section.

## Supporting information


**Supporting Information** The supporting information show the details of the questionnaires that were used in data collection from smallholder dairy cattle farmers (Section A), workers at milk collection centers (Section B) and Livestock/Veterinary Officers (Section C).

## Data Availability

Data are available on request from the authors.
