# Supplementary material for: Assessment of Dairy Cattle Management System and Uses of Antimicrobials Among Smallholder Farmers in Korogwe District, Tanzania
Source: Vet Med Int. 2026 Jun 26;2026:8596479. doi: 10.1155/vmi/8596479 (PMC13307180; doi:10.1155/vmi/8596479)
Supplement: Supplementary file 1 — Supporting Information The supporting information show the details of the questionnaires that were used in data collection from smallholder dairy cattle farmers (Section A), workers at milk collection centers (Section B) and Livestock/Veterinary Officers (Section C). [file VMI-2026-8596479-s001.doc]

**Supplementary materials**

**Dairy farmers’ questionnaire**

| **Introduction and informed consent**  Hello. My name is Goodluck Cleophas Mushi and I am working with the Local Government Authorities at Korogwe District Council, Tanga as a Senior Livestock Officer. I am conducting a survey study for assessing dairy famer’s awareness on the antimicrobial residues detected in marketed milk at Korogwe District Council.  I would very much appreciate your participation in this survey. I would like to ask you about some important information in dairy sector related to animal health and production, managerial practices, antimicrobial use, milking practices, access to information and resources. This information will help to assess presence of antimicrobial residues and milk safety quality assurances in food chain at Korogwe. I plan to collect milk samples from dairy cows and submit for laboratory diagnosis (Antimicrobial residuals detection Kit) and collect information for the risk factors which lead to antimicrobial use with associated consequences. It will take us about 30 minutes to complete the interview.    Whatever information you provide will be kept confidential and will not be shown to other people. Participation in this survey is voluntary, and you can choose not to answer any individual question or all the questions. However, we hope that you will participate in this survey since your opinions and information are important for development of dairy farming, public health protection of milk consumers and overall, Nation Health issues. At this time, do you want to ask me anything about the survey?  May I begin the interview now? [ ] Agree [ ] Disagree  If respondent agree to be interviewed . . . . . . . . .... 1 go continue Questioning  Respondent disagree to be interviewed …………2 ends (don’t continue Questioning)  Name of the enumerator: _________________________________________  Signature of Interviewer: _______________________Date: _____________________  Date checked by field supervisor: __________________  Supervisor’s Name______________________ Signature: __________________ |
| --- |

| **Demographic characteristics of respondents**  Questionnaire No. ________ Household No________ ID of respondent________________  Village/street ______________ Ward: _________________ District __________________  **Optional:**  Farmer/ Farm Name __________________Phone number: __________________  Note: ID represents the identification of the respondent (representative of the household who responds to the questionnaires). |
| --- |

**SECTION A: Smallholder dairy farmers**

A1. Socio-Demographic Information

Name of the respondent (optional): ___________________

Sex: ☐ Male ☐ Female

Age: ___________________ years

Education level:
☐ No formal education ☐ Primary ☐ Secondary ☐ Tertiary

Occupation (main): ___________________

Ward/Village: ___________________

A2. Herd and Milk Production Information

Number of cattle: __________________________________

Breeds are your cattle: ______________________________

Cattle management your cattle ☐ extensive, ☐ semi-intensive, ☐ extensive

Common diseases of cattle (please mention) (i)………………………………….

(ii)…………………………………………………. (iii)……………………………….

(iv)…………………………………………………. (v)……………….………………

Diseases control measures in place

☐ Dipping, ☐ vaccination, ☐ traditional uses of herbs, ☐ washing of animals with water, ☐ others (please mention) __________________________________________

Number of lactating cows currently owned: _________

Average milk yield per cow per day (litres): __________

Do you sell milk? ☐ Yes ☐ No

If yes, where do you sell your milk?
☐ Milk collection centre
☐ Vendors
☐ Households
☐ Restaurants/kiosks
☐ Others: _______________

A3. Antibiotic Use Practices

Do you use antibiotics in your cattle? ☐ Yes ☐ No

If yes, what type of antibiotics do you use?
☐ Tetracyclines ☐ Penicillins ☐ Sulfonamides ☐ Don’t know

What are the reasons of use

☐ Treatment, ☐ disease prevention, ☐ increase milk production, ☐ others (mention) _____________________________________________________________________

Who prescribes/administers the antibiotics?
☐ Self ☐ Agrovet ☐ Veterinarian ☐ Others: ___________

Do you consume or sell milk from a cow under antibiotic treatment? ☐ yes, ☐ No

Are you aware of the withdrawal period after treating with antibiotics? ☐ Yes ☐ No

Do you observe the withdrawal period before milking for consumption or sale? ☐ Always ☐ Sometimes ☐ Never

What do you do with milk during withdrawal period?
☐ Discard ☐ Use for household ☐ Feed to calves ☐ Sell

Have you ever been trained on proper drug use in animals? ☐ Yes ☐ No

**SECTION B: Milk collection centres**

B1. Centre Profile

Name of the milk collection centre: ______________________

Location (Ward/Village): ___________________________

Daily volume of milk collected (litres): __________

Number of regular milk suppliers: ___________

B2. Milk Quality Control Practices

Do you conduct any quality control tests on received milk? ☐ Yes ☐ No

If yes, which tests?
☐ Alcohol test ☐ Lactometer ☐ Antibiotic residue test ☐ Others: __________

Do you accept milk from cows under treatment or within withdrawal period? ☐ Yes ☐ No ☐ Not Sure

Are suppliers trained or informed about risks of drug residues in milk? ☐ Yes ☐ No

How frequent do you encounter milk of poor quality?

☐ Often, ☐ once a week, ☐ several times per week, ☐ rarely

What are the major causes of downgrading the quality of milk?

☐ Clotted milk, ☐ watery milk, ☐ dirty milk, ☐ milk with residue test, ☐ Others (*mention*): ______

What actions do you take when poor quality or contaminated milk is detected? (please mention) __

**SECTION C: Livestock/Veterinary Officers**

C1. Professional Profile

Name (optional): ___________________

Position/Title: _____________________

Ward/District assigned: __________________

Years of experience in livestock services: _______

C2. Drug Use and Surveillance

How frequently do you use antibiotics in dairy cattle in your area?
☐ Very frequent ☐ Frequent ☐ Rare ☐ Never

What are the commonly used antibiotics among farmers?
☐ Tetracyclines ☐ Sulfonamides ☐ Penicillins, ☐ Gentamycin, ☐ Others (mention): __________________________________________________

Reasons for using antibiotics in dairy cattle? ☐ Treatment, ☐ disease prevention, ☐ increase milk production, ☐ others (mention) ___________________________________________________________________

Do you give instructions to farmers on withdrawal period of milk from cows under antibiotic treatments? ☐ Yes, ☐ No

Are farmers generally aware of withdrawal periods? ☐ Yes, ☐ No

Do you conduct training/sensitization on drug use and withdrawal periods? ☐ Yes ☐ No

Are there any reported cases of drug residues in milk in your area? ☐ Yes ☐ No

What monitoring mechanisms are in place to check for antibiotic residues in milk?

**SECTION D: Veterinary shop dealers**

D1. Business Profile

Name of Agrovet shop: ___________________________

Location (Ward/Village): _________________________

Qualifications of the owner/operator:
☐ Veterinarian ☐ Animal Health Technician ☐ Businessperson ☐ Others

Do you sell antibiotics to livestock owners? ☐ Yes, ☐ No

D2. Antibiotic Sales and Awareness

Which antibiotics are most frequently sold for use in dairy cattle?
☐ Tetracyclines ☐ Penicillins ☐ Sulfonamides, ☐ Gentamycin, ☐ others (mention)___________________________________________________

Do you require a prescription before antibiotic sale? ☐ Always ☐ Sometimes ☐ Never

Do you provide information about withdrawal periods to clients? ☐ Yes ☐ No

Are you aware of the health risks of antibiotic residues in milk? ☐ Yes ☐ No

If yes, mention the health risks of antibiotic residues in milk (i)………………….

(ii)……………………………………(iii)……………………………. (iv)……………

Have you received any training on prudent antibiotic use? ☐ Yes ☐ No
